# Personality Traits and Teaching Commitment Among Pre-Service Teachers: Teaching Motivation as a Mediator

**DOI:** 10.3390/bs15040548

**Published:** 2025-04-18

**Authors:** Jie Liu, Qingxi Yang, Jin Yang, Shu Wang, Hongbiao Yin

**Affiliations:** 1 Faculty of Education, Northeast Normal University, Renmin Street 5268, Changchun 130021, China; liujie@nenu.edu.cn (J.L.); yangqx779@nenu.edu.cn (Q.Y.); yangj614@nenu.edu.cn (J.Y.); 2 Department of Curriculum & Instruction, Faculty of Education, The Chinese University of Hong Kong, Shatin, N.T., Hong Kong; yinhb@cuhk.edu.hk

**Keywords:** HEXACO traits, teaching commitment, pre-service teachers, motivation

## Abstract

With the HEXACO model of personality, this study examined the relationship between HEXACO traits (i.e., honesty–humility, emotionality, extraversion, agreeableness, conscientiousness, and openness to experience) and teaching commitment among 2031 Chinese pre-service teachers. The results show that all HEXACO traits positively predicted teaching commitment, with extraversion and agreeableness being the most pronounced predictors. Also, this study investigated whether teaching motivation, including intrinsic motivation, altruistic motivation, and extrinsic motivation, could explain the relationship between HEXACO traits and teaching commitment. The results supported the mediational role of teaching motivation, with intrinsic motivation showing the strongest mediational effect. For extraversion and openness to experience, a full mediation model was supported, while for the other four HEXACO traits, a partial mediation model was validated. The theoretical and practical implications of this study for teacher education are discussed.

## 1. Introduction

Teachers’ decisions to enter or stay in the teaching profession have triggered a great interest among researchers, and are typically termed teaching commitment (Chesnut & Cullen, 2014; R. M. Klassen & Chiu, 2011). Among pre-service teachers, teaching commitment refers to one’s decisions concerning preparing for and/or choosing teaching as a future career (R. M. Klassen & Chiu, 2011; Rots et al., 2007).

Some research has already demonstrated the importance of teaching commitment for pre-service teachers. For example, teaching commitment is reported to be associated with greater confidence about teaching (Chesnut & Burley, 2015), higher devotion to teacher-training programs (Rots et al., 2007), more engagement in organization citizenship behaviors (Somech & Bogler, 2002), and better achievements of their students (Chesnut & Cullen, 2014). Additionally, for pre-service teachers who are more committed to the teaching profession, they tend to be more satisfied with their choice of being a teacher, work harder in teaching preparation, and stay longer in the teaching profession (Thomson & Palermo, 2014).

Accordingly, it is critical to examine factors influencing pre-service teachers’ teaching commitment. One such factor might be pre-service teachers’ personality traits, which are defined as individual differences in their general tendency to behave, think, or feel (Ashton, 2013). Indeed, personality traits are associated with various important life outcomes, such as health (Sutin et al., 2016), career interest (Lee et al., 2022) and job performance (Dudley et al., 2006). Thus, it is plausible to assume that personality traits are associated with teaching commitment.

This study relies on the HEXACO model of personality (Lee & Ashton, 2008), comprising six personality traits: honesty–humility, emotionality, extraversion, agreeableness, conscientiousness, and openness to experience. Honesty–humility is characterized by being honest, sincere and fair versus being deceiving, manipulative and greedy. Emotionality is characterized by being sentimental, anxious and dependent versus being brave, tough and independent. Extraversion is characterized by being outgoing, talkative and cheerful versus being shy, quiet and reserved. Agreeableness is characterized by being forgiving, lenient and patient versus being quarrelsome and stubborn. Conscientiousness is characterized by being organized, diligent and prudent versus being irresponsible, lazy and sloppy. Openness to experience is characterized by being curious, creative and imaginative versus being shallow and conventional.

The HEXACO model of personality has been shown to be advantageous compared to other personality models, such as the Five-Factor Model (FFM; Goldberg, 1990; McCrae & Costa, 2003). Firstly, compared to FFM, which was developed with a lexical analytical approach in the English language, the HEXACO model of personality was developed and validated in more than ten languages (Lee & Ashton, 2008). Secondly, the HEXACO model of personality has been shown to have incremental predictive validity to various outcome criteria compared to FFM, such as counterproductive work behaviors (Pletzer et al., 2019), self-centered behaviors (De Vries et al., 2009), and vocational interest (McKay & Tokar, 2012). Recently, some studies have further confirmed the theoretical and empirical advantages of the HEXACO model of personality (Zettler et al., 2020).

Particularly, the addition of honesty–humility in the HEXACO model makes it more suitable to examine teaching commitment in Chinese culture. Honesty–humility is highly related to morality and ethical standards (Howard & Van Zandt, 2020), and thus could reflect the morality of a person. Since the Chinese culture highlights the importance of teachers being moral models to their students, this trait might be particularly important for teaching commitment. However, no study to date has examined pre-service teachers’ teaching commitment based on the HEXACO model of personality. Thus, this study aims to examine the relationship between HEXACO traits and teaching commitment to not only shed some light on the relationship between honesty–humility and teaching commitment, but also extend the application of the HEXACO model of personality in the field of teacher education.

Though no study has directly investigated the relationship between HEXACO traits and teaching commitment, some indirect suggestions can be gleaned from the literature examining related topics. For example, students rated “fair, just, impartial, and have no pets or favorites” more frequently as important qualities that should be possessed by teachers (Jersild, 1940). These characteristics nicely mirror features of honesty–humility. As teaching professions involve frequent social interactions (Pyhältö et al., 2011), extraversion-related characteristics are likely to facilitate these tasks. The ability to manage competing priorities, be highly organized and effective with time, termed “organization and planning”, are also important characteristics for teachers (R. Klassen et al., 2016). These features perfectly echo the characteristics of conscientiousness.

The association between personality traits and life outcomes is likely to be interpreted by factors particularly related to specific life domains (McAdams & Pals, 2006). For example, Bahrami and Hosseini (2023) found that the relationship between personality traits and teacher research involvement was mediated by motivation related to conducting research among language teachers. Relating to our research, teaching motivation is likely to mediate the relationship between personality traits and teaching commitment.

Teaching motivation is typically categorized into three types, including altruistic, intrinsic, and extrinsic motivation (Fray & Gore, 2018). Altruistic motivation is characterized by “service to others” (Osguthorpe & Sanger, 2013). Helping and supporting children (Jungert et al., 2014), answering a call (Osguthorpe & Sanger, 2013), and making social contribution (Flores & Niklasson, 2014) are some altruistic reasons that are frequently reported by pre-service teachers. Intrinsic motivation is related to teaching itself and defined as inherent satisfaction from teaching-related activities (Thomson & Palermo, 2014). There are various intrinsic reasons for teaching, including interest in and enjoyment of teaching (Mtika & Gates, 2011), being suited to teaching career (Chong & Low, 2009), and liking working with children or adolescents (Flores & Niklasson, 2014). Extrinsic motivation is characterized by obtaining satisfaction from external rewards (Fray & Gore, 2018). For the teaching profession, some commonly mentioned external rewards include flexible working hours, lengthy holidays, job security, and good salaries (Aksu et al., 2010; Cheung & Yuen, 2016; Jungert et al., 2014).

To sum up, the current study examines the relationship between HEXACO traits, teaching motivation and teaching commitment among Chinese pre-service teachers. Specifically, we have two research questions:

(1) How do Chinese pre-service teachers’ HEXACO personality traits affect their teaching commitment?

(2) How does teaching motivation mediate the relationship between HEXACO traits and teaching commitment?

## 2. Methods

### 2.1. Participants and Procedures

This study was conducted online and we mainly used a convenience sampling method to recruit participants. The online survey was shared by our coauthors with several universities that mainly educate teachers in the northeast of China. The participants were informed in the first page of the online survey that their participation was voluntary and anonymous, and they could drop out of the survey anytime they want. The participants were also told that they would receive personalized personality feedback after completing the online survey. In total, 2084 participants answered our online survey, and 53 responses were omitted due to incomplete data, resulting in a sample comprising 2031 participants (1784 female). The participants were aged between 17 and 31 (*M* = 19.52, *SD* = 1.55), 40.7% majored in elementary education, 35.5% majored in pre-school education, 15% majored in general education, and the rest majored in other educational disciplines, such as art education, science education, and technical education.

### 2.2. Measures

Personality traits: Personality traits were measured with the HEXACO-60 (Ashton & Lee, 2009), measuring each trait with 10 items. These items were answered with a 5-point Likert scale (1 = strongly disagree; 5 = strongly agree). HEXACO-60 has been used in Chinese population, and has demonstrated good reliability (Liu et al., 2018). The item order was randomized for each participant. The reliabilities for the HEXACO traits ranged from 0.65 to 0.77.

Teaching motivation: Teaching motivation was measured with 10 subscales from the FIT-choice scale (Watt & Richardson, 2007), including intrinsic value (0.86), working with children/adolescents (0.90), perceived teaching abilities (0.83), shape the future of children/adolescents (0.85), enhance social equity (0.89), make a social contribution (0.91), social status (0.87), income (0.86), security (0.87), and time for family (0.78). These items were answered with a 7-point Likert scale (1 = strongly disagree; 7 = strongly agree). The reliabilities of these dimensions from FIT-choice were presented in the bracket.

Teaching commitment: Teaching commitment was measured by three items assessing one’s willingness to choose teaching professions in the future. These items were answered with a 7-point Likert scale (1 = strongly disagree; 7 = strongly agree). The reliability of teaching commitment was 0.86.

### 2.3. Analytical Strategies

All the analyses were conducted in R 4.4.0. To begin, we evaluated the three-factor teaching motivation structure, i.e., intrinsic, altruistic and extrinsic motivation, with structural equation modeling (SEM). We performed SEM with the lavaan package 0.6-17 (Rosseel, 2012).

In particular, intrinsic motivation comprised intrinsic value, work with children/adolescents and perceived teaching abilities. Altruistic motivation comprised shape the future of children/adolescents, enhance social equality, and make a social contribution. Extrinsic motivation comprised social status, salary, job security, and time for family. According to the suggested criteria that values of TLI ≥ 0.90, CFI ≥ 0.90 and RMSEA ≤ 0.60 indicate a good model fit (Kline, 2016), the fit values of our model are as follows: CFI = 0.929, ILI = 0.922, RMSEA = 0.058 (90% CI [0.056, 0.059]), indicating that our model was acceptable. Thus, we used these three motivation factors, i.e., intrinsic, altruistic, and extrinsic motivation in the following analyses. Also, the assumptions for regression and mediation analyses were met in our study.

## 3. Results

### 3.1. The Relationship Between HEXACO Traits and Teaching Commitment

Table 1 shows the mean, standard deviation and reliability of each variable involved in this study as well as zero-order correlations among these variables. The correlations between HEXACO traits and teaching commitment ranged from 0.13 to 0.25 (*p*s < 0.01). The relationship of altruistic, intrinsic, and extrinsic motivation and teaching commitment ranged from 0.47 to 0.72 (*p*s < 0.01).

For research question 1 concerning the relationship between each HEXACO trait and teaching commitment, linear regression models were used to predict teaching commitment from each of the HEXACO traits. The results are presented in Table 2, showing that all HEXACO traits positively and significantly predicted teaching commitment (0.30 ≤ *β* ≤ 0.54, *p*s < 0.001), with extraversion being the strongest predictor and openness to experience being the weakest predictor.

### 3.2. Testing Teaching Motivation as a Mediator

Research question 2 aimed to test whether teaching motivation mediates these relations. For the correlation between each HEXACO trait and teaching commitment, a mediation model with teaching motivation (i.e., intrinsic, altruistic, and extrinsic motivation) as a mediator was performed using the “mediate” function, which produces 95% confidence intervals for the indirect effects using 5000 resamples, in the “psych” package in R (Tingley et al., 2014). Please note that the three types of teaching motivation were entered simultaneously into the mediation models to account for any overlapping among them. All results are presented in Table 2, and below we describe each model in more detail.

**Figure 1:**
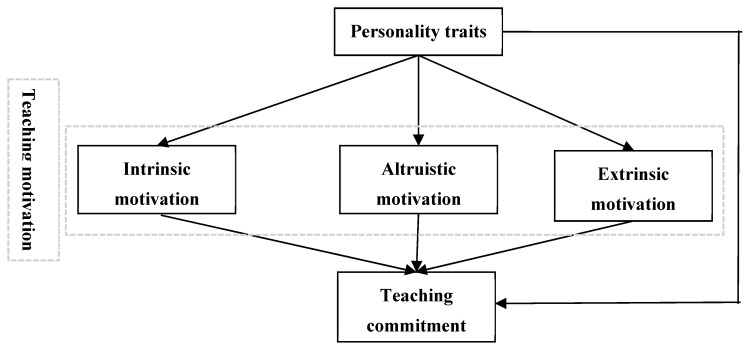
Teaching motivation (i.e., intrinsic motivation, altruistic motivation, extrinsic motivation) mediated the relationship between personality traits and teaching commitment.

The proposed mediation model is depicted in Figure 1, illustrating that teaching motivation (i.e., intrinsic, altruistic, and extrinsic motivation) mediates the relationship between HEXACO traits and teaching commitment. We firstly tested this model with honesty–humility. To test whether teaching motivation mediates the relationship between honesty–humility and teaching commitment, we used honesty–humility to predict three teaching motivations, and the results show that honesty–humility significantly predicted the three teaching motivations (−0.06 ≤ *β* ≤ 0.51, *p*s ≤ 0.045). Then, we predicted teaching commitment from honesty–humility and teaching motivation, and the results indicate that both predictors can significantly predict teaching commitment (0.07 ≤ *β* ≤ 0.60, *p*s ≤ 0.016). The mediation model shows that the indirect effects were 0.27 (95% CI [0.21, 0.34]), 0.04 (95% CI [0.01, 0.07]), and −0.02 (95% CI [−0.04, 0]) for intrinsic, altruistic and extrinsic motivation, respectively, and the total indirect effect was 0.29 (95% CI [0.22, 0.36]) for teaching motivation.

The same method was used to test the mediation effect of teaching motivation on the other traits and teaching commitment, and the results indicate that the indirect effects of teaching motivation was 0.20 (95% CI [0.11, 0.27]) for emotionality and teaching commitment, 0.52 (95% CI [0.45, 0.59]) for extraversion and teaching commitment, 0.41 (95% CI [0.32, 0.49]) for agreeableness and teaching commitment, 0.32 (95% CI [0.23, 0.40]) for conscientiousness and teaching commitment, and 0.36 (95% CI [0.29, 0.44]) for openness to experience and teaching commitment. Noticeably, for extraversion and openness to experience, a full mediation model was validated, and for the rest, a partial mediation model was established.

## 4. Discussion

This study examined the relationship between HEXACO personality traits, teaching motivation and teaching commitment among Chinese pre-service teachers. We found that all HEXACO traits positively predicted teaching commitment, with extraversion being the strongest predictor, and teaching motivation, including intrinsic, altruistic, and extrinsic motivation, mediates these relations. These results indicate that pre-service teachers’ personality traits are associated with their future career choice, and teaching motivation could explain these relations.

### 4.1. HEXACO Traits and Teaching Commitment

Our study helps to depict personal characteristics of a committed teacher from an established personality model, showing that a committed pre-service teacher has higher levels of all HEXACO traits. Our effort nicely answers the call from Göncz (2017), stating that more studies relying on established personality frameworks should be conducted given the shortage of such research. In addition, the conclusion that HEXACO personality traits are associated with pre-service teachers’ commitment to their future occupations aligns quite well with previous studies suggesting that personality traits are predictive of the more broadly defined occupational commitment (Choi et al., 2015). Accordingly, our study not only helps to bridge the communication between researchers from personality psychology and teacher education, but also extend the application of the HEXACO model.

Extraversion and agreeableness are the most pronounced predictors of teaching commitment. That is to say, being extraverted and agreeable are more important for a teacher to be committed to the teaching profession. This might be because these two traits are critical for jobs requiring interpersonal interactions (Mount et al., 1998), and are beneficial in establishing good teacher–student relationships (Kim & MacCann, 2018). Given the fundamental social nature of the teaching profession, being extraverted and agreeable are likely to help teachers to fulfil two primary components of the teaching profession, providing instructional and emotional support to students.

Noticeably, we are the first to establish the relationship between honesty–humility and teaching commitment, and find that pre-service teachers high in honesty–humility tend to be more loyal to the teaching profession. Thus, honesty–humility plays an important role in determining pre-service teachers’ teaching commitment. This might be due to the educational policies in China, particularly emphasizing morality in teacher education. Classes like moral education are compulsory for every teacher student and characteristics associated with morality, such as fairness, honesty, and modesty, are highly advocated. In addition, Confucianism, a guiding philosophy for Chinese society for over two millennia, particularly highlights morality, justice, and ethical standards in teachers. Additionally, China issued a landmark document in late August in 2024 to stress teachers’ moral standards as one of the most important characteristics that should be cultivated in pre-service teachers. Future research should continue examining the relationship between honesty–humility and teaching commitment in different cultures to test the generalization of our conclusions in other cultures.

### 4.2. The Mediational Role of Teaching Motivation

Intrinsic, altruistic, and extrinsic motivation have different mediational effects on the relationships between HEXACO traits and teaching commitment, with intrinsic motivation carrying the largest indirect effect. Theoretically speaking, this result aligns quite well with the gist of self-determination theory (Ryan & Deci, 2020), ranking intrinsic motivation as the highest form of self-determined motivation and emphasizing the significance of intrinsic motivation in directing one’s behaviors. Empirically speaking, some studies have shown that interest in teaching is the most important reason for choosing the teaching profession (Chong & Low, 2009; Fray & Gore, 2018).

For the relationships between teaching commitment and honesty–humility, emotionality, agreeableness, and conscientiousness, teaching motivation has a partial mediational effect. This suggests the existence of other potential mediators, and future research could continue examining this. As honesty–humility, emotionality and agreeableness are mainly about initiating cooperation and maintaining interpersonal relationships (Ashton & Lee, 2007), perhaps interpersonal attachment or belongingness could be potential mediators. It is likely that pre-service teachers with higher levels of these traits might feel a sense of belonging to the teaching profession or psychologically attached to their students, and thus are more committed to teaching. For conscientiousness, the preference for order and predictability might make the teaching profession more appealing. That is to say, the reason why pre-service teachers high in conscientiousness are more committed to teaching might be partially because they like the order and structure associated with the teaching profession. Future studies could continue examining these potential mediators. For extraversion and openness to experience, their relationships with teaching commitment are fully accounted for by teaching motivation. This result indicates that for pre-service teachers high in extraversion or openness to experience, the reason why they choose to become a teacher is because that they like teaching, want to contribute to society, and value the external rewards associated with being a teacher. Particularly, the reason why pre-service teachers high in these two traits are more committed to teaching is primarily driven by intrinsic motivation. Concerning openness to experience, this result perfectly echoes conclusions from Bahrami and Hosseini (2023), documenting significant associations between openness to experience and intrinsic motivation for development. Since teaching offers opportunities for life-long learning and personal development, it perfectly matches the behavioral patterns of people high in openness to experience—pursing knowledge and satisfying curiosity. Concerning extraversion, the teaching profession ideally gratifies the ultimate goal pursued by extraverts—gaining social attention (Ashton et al., 2002). Being a teacher is somewhat like being a leader, being admired and respected by students. Accordingly, the relationship between these two traits and teaching commitment is fully explained by teaching motivation. Future research could examine some moderators that could influence these relations.

### 4.3. Implications and Limitations of This Study

This study has some implications for teaching and teacher education. Firstly, since using personality traits to screen suitable candidates is quite common in the workplace (Salgado & De Fruyt, 2017), possibly using personality tests to screen potentially committed pre-service teachers is promising in teacher education. Since teacher shortage is a problem worldwide, using personality traits to screen potentially committed teacher candidates might help to mitigate this problem. However, using personality traits to make high-state decisions (e.g., hiring or recruiting) could trigger some unexpected behaviors, such as cheating or faking. Perhaps teacher education programs could consider creating personality archives for each pre-service teacher when entering this program. That is to say, personality traits of each pre-service teacher should be assessed not only in the beginning of the teacher education program, but also consistently during the process, perhaps once per year. In this case, the likelihood of cheating would be lower compared to the case of assessing personality traits at one point. With such personality archives, suitable teacher candidates should be encouraged to pursue teaching as their career, whereas those who are not suitable to be teachers could be encouraged to seek other job opportunities. However, creating personality archives is just a speculation, and future research could examine the feasibility of such a method. In addition, an alternative to applying personality trait screening in teacher education is to provide teacher candidates with relevant training. For example, practitioners can offer personality education to teacher students, help them to have a better understanding of their own personalities, to know the important personality traits for being an effective teacher, and to motivate students to intentionally behave in ways that breed these preferred personality traits. As personality traits are changeable, though modestly, via interventions (Roberts et al., 2017), training is likely to be beneficial in the long run.

The conclusion that intrinsic motivation accounts for the largest variances in the relationships between HEXACO traits and teaching commitment implies that increasing intrinsic motivation is critical to enhance teaching commitment. Offering positive feedback (Fong et al., 2019), encouraging autonomy (Ryan & Deci, 2020), focusing on developing competence rather than acquiring performance (Urdan & Kaplan, 2020), and providing relatedness support (Ahmadi et al., 2023) are good ways to foster intrinsic teaching motivation among pre-service teachers. Though to a lesser degree, altruistic and extrinsic motivation also explain some variance in the relationships between HEXACO traits and teaching commitment. These results suggest that creating an environment facilitating cooperation and guaranteeing material rewards are important for obtaining and retaining teachers.

The current study also has some limitations. Firstly, since our participants are pre-service teachers, this prevents the generalization of our conclusion to other groups, such as in-service teachers. Future research could examine whether our conclusions are still valid among in-service teachers. Secondly, we have not examined the influence of other teaching-related factors, such as teaching phases and teaching subjects. Future research could take these factors into consideration. Thirdly, as teaching commitment is likely to change over time, future research could examine the dynamic processes between personality traits and teaching commitment with more advanced methods, such as longitudinal study designs or experimental interventions. Additionally, the three-component model of commitment has pointed out that commitment is a multidimensional construct, comprising affective commitment, continuance commitment, and normative commitment (Allen & Meyer, 1990). Future studies could examine teaching commitment from these three aspects to further reveal the relationship between personality traits and different aspects of teaching commitment. Finally, our study was conducted in China, and future studies could continue examining the relationship between HEXACO traits and teaching commitment in other cultures to shed some light on whether culture has an impact on these relations.

## Figures and Tables

**Table 1:** Means, standard deviations, and zero-order correlations among main variables.

Variable	*M*	*SD*	1	2	3	4	5	6	7	8	9	10
1. Honesty–humility	3.36	0.63	0.73									
2. Emotionality	3.45	0.58	−0.10 **	0.71								
3. Extraversion	3.26	0.62	0.08 **	−0.12 **	0.77							
4. Agreeableness	3.27	0.53	0.37 **	−0.17 **	0.30 **	0.65						
5. Conscientiousness	3.18	0.52	0.25 **	−0.04	0.24 **	0.22 **	0.66					
6. Openness to experience	3.29	0.57	0.11 **	−0.08 **	0.27 **	0.21 **	0.19 **	0.66				
7. Intrinsic motivation	5.09	1.21	0.24 **	0.10 **	0.33 **	0.24 **	0.17 **	0.22 **	-			
8. Altruistic motivation	5.42	1.21	0.27 **	0.15 **	0.30 **	0.25 **	0.19 **	0.26 **	0.81 **	-		
9. Extrinsic motivation	4.75	0.89	−0.04 *	0.11 **	0.19 **	0.06 **	0.07 **	0.05 *	0.46 **	0.49 **	-	
10. Teaching commitment	5.33	1.31	0.22 **	0.13 **	0.25 **	0.21 **	0.17 **	0.14 **	0.72 **	0.63 **	0.47 **	0.86

Note. * *p* < 0.05. ** *p* < 0.01. Cronbach’s αs are presented in the diagonal.

**Table 2:** The mediation role of teaching motivation in explaining the relationship between HEXACO traits and teaching commitment.

**Honesty** **–Humility Model**					**Emotionality Model**				
**Paths: HH → Teaching Motivation → Teaching Commitment**					**Paths: EM → Teaching Motivation → Teaching Commitment**				
HH → Teaching Commitment	*β*	*SE*	*t*	*p*	EM → Teaching Commitment	*β*	*SE*	*t*	*p*
HH	0.46	0.04	10.38	<0.001	EM	0.30	0.05	6.06	<0.001
HH → Teaching motivation	*β*	*SE*	*t*	*p*	EM → Teaching motivation	*β*	*SE*	*t*	*p*
Intrinsic motivation	0.45	0.04	10.96	<0.001	Intrinsic motivation	0.21	0.05	4.51	<0.001
Altruistic motivation	0.51	0.04	12.40	<0.001	Altruistic motivation	0.30	0.05	6.63	<0.001
Extrinsic motivation	−0.06	0.03	−2.01	0.045	Extrinsic motivation	0.17	0.03	5.13	<0.001
HH + Teaching motivation → Teaching Commitment	*β*	*SE*	*t*	*p*	EM + Teaching motivation → Teaching Commitment	*β*	*SE*	*t*	*p*
HH	0.17	0.03	5.25	<0.001	EM	0.10	0.03	3.04	0.002
Intrinsic motivation	0.60	0.03	21.60	<0.001	Intrinsic motivation	0.62	0.03	21.98	<0.001
Altruistic motivation	0.07	0.03	2.41	0.016	Altruistic motivation	0.09	0.03	3.07	0.002
Extrinsic motivation	0.27	0.03	10.45	<0.001	Extrinsic motivation	0.24	0.03	9.33	<0.001
Indirect effects	*Est.*		95% CI		Indirect effects	*Est.*	95% CI		
Intrinsic motivation	0.27		[0.21, 0.34]		Intrinsic motivation	0.13	[0.06, 0.19]		
Altruistic motivation	0.04		[0.01, 0.07]		Altruistic motivation	0.03	[0.01, 0.05]		
Extrinsic motivation	−0.02		[−0.04, 0]		Extrinsic motivation	0.04	[0.02, 0.06]		
Total indirect effects	0.29		[0.22, 0.36]		Total indirect effects	0.20	[0.11, 0.27]		
**Extraversion model**					**Agreeableness model**				
**Paths: EX → Teaching motivation → Teaching commitment**					**Paths: AG → Teaching motivation → Teaching commitment**				
EX → Teaching Commitment	*β*	*SE*	*t*	*p*	AG → Teaching Commitment	*β*	*SE*	*t*	*p*
EX	0.54	0.05	11.82	<0.001	AG	0.51	0.05	9.49	<0.001
EX → Teaching motivation	*β*	*SE*	*t*	*p*	AG → Teaching motivation	*β*	*SE*	*t*	*p*
Intrinsic motivation	0.65	0.04	15.79	<0.001	Intrinsic motivation	0.54	0.50	10.92	<0.001
Altruistic motivation	0.59	0.04	14.34	<0.001	Altruistic motivation	0.57	0.05	11.55	<0.001
Extrinsic motivation	0.27	0.03	8.75	<0.001	Extrinsic motivation	0.11	0.04	2.81	0.005
EX + Teaching motivation → Teaching Commitment	*β*	*SE*	*t*	*p*	AG + Teaching motivation → Teaching Commitment	*β*	*SE*	*t*	*p*
EX	0.02	0.03	0.51	0.613	AG	0.11	0.04	2.74	0.006
Intrinsic motivation	0.61	0.03	21.52	<0.001	Intrinsic motivation	0.61	0.03	21.62	<0.001
Altruistic motivation	0.10	0.03	3.34	0.001	Altruistic motivation	0.09	0.03	3.04	0.002
Extrinsic motivation	0.24	0.03	9.46	<0.001	Extrinsic motivation	0.25	0.03	9.69	<0.001
Indirect effects	*Est.*		95% CI		Indirect effects	*Est.*	95% CI		
Intrinsic motivation	0.40		[0.33, 0.47]		Intrinsic motivation	0.33	[0.26, 0.41]		
Altruistic motivation	0.06		[0.01, 0.10]		Altruistic motivation	0.05	[0.01, 0.09]		
Extrinsic motivation	0.07		[0.05, 0.09]		Extrinsic motivation	0.03	[0.01, 0.05]		
Total indirect effects	0.52		[0.45, 0.59]		Total indirect effects	0.41	[0.32, 0.49]		
**Conscientiousness model**					**Openness to experience model**				
**Paths: CO → Teaching motivation → Teaching commitment**					**Paths: OP → Teaching motivation → Teaching commitment**				
CO → Teaching Commitment	*β*	*SE*	*t*	*p*	OP → Teaching Commitment	*β*	*SE*	*t*	*p*
CO	0.43	0.05	7.77	<0.001	OP	0.32	0.05	6.30	<0.001
CO → Teaching motivation	*β*	*SE*	*t*	*p*	OP → Teaching motivation	*β*	*SE*	*t*	*p*
Intrinsic motivation	0.41	0.05	8.01	<0.001	Intrinsic motivation	0.47	0.05	10.09	<0.001
Altruistic motivation	0.45	0.05	8.91	<0.001	Altruistic motivation	0.57	0.05	12.35	<0.001
Extrinsic motivation	0.12	0.04	3.16	0.002	Extrinsic motivation	0.07	0.04	2.12	0.034
CO + Teaching motivation → Teaching Commitment	*β*	*SE*	*t*	*p*	OP + Teaching motivation → Teaching Commitment	*β*	*SE*	*t*	*p*
CO	0.11	0.04	2.83	0.005	OP	−0.04	0.04	−1.19	0.236
Intrinsic motivation	0.61	0.03	21.76	<0.001	Intrinsic motivation	0.61	0.03	21.85	<0.001
Altruistic motivation	0.09	0.03	3.10	0.002	Altruistic motivation	0.10	0.03	3.53	<0.001
Extrinsic motivation	0.24	0.03	9.59	<0.001	Extrinsic motivation	0.24	0.03	9.32	<0.001
Indirect effects	*Est.*		95% CI		Indirect effects	*Est.*	95% CI		
Intrinsic motivation	0.25		[0.18, 0.32]		Intrinsic motivation	0.29	[0.23, 0.35]		
Altruistic motivation	0.04		[0.01, 0.08]		Altruistic motivation	0.06	[0.02, 0.10]		
Extrinsic motivation	0.03		[0.01, 0.05]		Extrinsic motivation	0.02	[0, 0.04]		
Total indirect effects	0.32		[0.23, 0.40]		Total indirect effects	0.36	[0.29, 0.44]		

Note. HH = Honesty–humility, EM = Emotionality, EX = Extraversion, AG = Agreeableness, CO = Conscientiousness, OP = Openness to Experience. CI = Confidence interval.

## Data Availability

The data that support the findings of this study are available upon request from the corresponding author.
